# Teachers' perspectives on the webex online platform for secondary education during the COVID-19 pandemic in Greece

**DOI:** 10.1016/j.heliyon.2024.e39175

**Published:** 2024-10-22

**Authors:** Petros Violakis, Tilemachos Tzakopoulos

**Affiliations:** a Homeland Security, Rabdan Academy, Abu Dhabi, United Arab Emirates; b Independent Researcher, Omiros Educational Group, Thessaloniki, Greece

**Keywords:** Information systems (IS), COVID-19 pandemic, Webex platform, Greek educational system, Distance learning, Organisational performance, Secondary education, Technological developments

## Abstract

In this study, we examine the impact of Information Systems (IS) on the COVID-19 pandemic, focusing on the utilisation of the "Webex" online platform within the Greek educational system. This assessment aims to evaluate the platform's impact on organisational performance within this industry, particularly focusing on teachers' perspectives. This paper presents original quantitative research exploring how technological advancements have facilitated distance learning driven by the COVID-19 pandemic. The research is important because it highlights the **challenges** faced by Greece, as shown by the Digital Economy and Society Index (DESI) indicators [1]. In 2019, Greece ranked 26th out of 28 Member States, indicating outdated infrastructure and a lack of training among educators. Additionally, the country experienced a "brain drain" as a result of the 2008 Greek financial crisis, leading to skilled educators leaving the country [2]. The adoption of distance learning at the secondary education level in Greece presents unique challenges as well as opportunities, according to the research. The **novelty** of this study is its focus on primary data gathered from secondary school teachers in relation to the country's technological and other challenges. It provides insights into the real-world application and effectiveness of the Webex platform in an unprecedented situation. The study **contributes** to new knowledge by providing a detailed understanding of teachers' IT competency, their perceptions of online education, and the state's support (or lack thereof). It also highlights the psychological benefits of online education for students despite the noted challenges in attendance and achieving educational objectives. However, these lessons were not consistent for all students. Based on the statistical analysis, factors such as attendance, challenges perceived by the students, and the achievement of educational objectives were determined to be indicators of the effectiveness of online instructors. **Practically**, this research highlights the necessity for improved training for teachers to effectively utilise online platforms. It also **recommends** enhancing IT infrastructure and government support to improve online education. Teachers expressed openness to training seminars and recognised the psychological gains for students, suggesting the potential for continued use of online teaching methods post-pandemic. The wider sociopolitical impact involves informing discussions on the necessity, value, and effectiveness of e-learning, thus guiding future educational policies and practices.

## 1 The rationale for the research

### 1.1 Introduction

Education stands as a cornerstone of modern society, pivotal in nurturing individuals' intellectual, social, and personal growth. As societies evolve, so do the methodologies employed in the educational process, adapting to the dynamic needs and demands of the time [3]. In recent decades, technological advancements have revolutionised various sectors, including education, offering new tools and methods to enhance learning experiences. Online education, once primarily associated with higher education and lifelong learning seminars, has progressively become a significant component of educational systems worldwide.

However, in many countries, including Greece, primary and secondary education have traditionally relied on the physical presence of students and teachers. This conventional approach stems from the belief that distance learning cannot fully replicate the pedagogical and social aspects of in-person education [4]. Furthermore, Greece has faced challenges such as a lack of digital infrastructure and limited technology skillsets among educators and students and also because of the "brain drain" effect of the 2008 Greek ecenomic crisis [[2], [5],^6^].

The COVID-19 pandemic, which necessitated social distancing, abruptly forced educational institutions to adopt online teaching methods across all levels of education [7]. This sudden shift presented significant challenges, particularly in Greece, where both students and teachers were largely unprepared for the transition to digital learning environments. It is estimated that over 1.6 billion students globally were affected by the pandemic's impact on education [8]. In Greece, the pandemic highlighted the need to explore the implications of this new reality, especially given the country's reliance on traditional educational methods and the minimal integration of digital tools [1,9,10].

Despite the global shift towards online education, there remains a paucity of research specifically examining the effectiveness of platforms like Webex in the context of Greek secondary education during the pandemic. While existing studies have addressed various aspects of online learning, few have focused on the unique challenges and opportunities presented by the rapid transition to distance learning in a technologically underprepared environment like Greece [1,[10], [13]]. In Greece, technology is not widely used in the educational process, and teachers, as well as students, are not familiar with IT teaching processes. It is interesting to explore the aspects of this new reality. Programs like "e-omogeneia," "oikade," or "e-Hermes" are examples of distance learning in secondary education, but they have limitations [9].

This study aims to fill the gap in empirical data on the effectiveness of e-learning platforms, with a specific focus on the use of Webex in Greek secondary education. The study will investigate the impact of the Webex platform, which was widely utilized during the quarantine period from March to June 2020, on Greek secondary education. The study will highlight both the obstacles encountered and the psychological benefits observed.

By focusing on teachers' perspectives, this research seeks to understand their competency in using the online environment, their recognition of the importance of online education, and the role of the state in supporting this transition [[14], [15], [16]]. Additionally, the study will evaluate the effectiveness of online education in achieving educational objectives and identify the challenges faced by both teachers and students during this unprecedented period [[17], [18], [19]].

By addressing these research questions, the study aims to contribute valuable insights into the implementation and effectiveness of distance learning, particularly in the context of emergencies like the COVID-19 pandemic. The findings are expected to inform future educational policies and practices, ensuring that online teaching methods can be effectively integrated and supported, thus enhancing the overall educational experience in Greece and beyond.

### 1.2 Research gap

Despite the global shift towards online education, there is a lack of specific research on the effectiveness of the Webex platform in the context of Greek secondary education during the COVID-19 pandemic. While previous studies have explored different facets of online learning, only a few have specifically addressed the challenges and opportunities arising from the sudden shift to distance learning in a technologically unprepared setting such as Greece. This study aims to fill this gap by providing empirical data on the effectiveness of Webex in Greek secondary education, highlighting both the obstacles encountered and the psychological benefits observed.

Bearing the above in mind, it is interesting to reveal the different aspects of the implementation of distant learning, especially in urgent situations like the one created by the COVID-19 pandemic. The present study can be a reference point for reaching important conclusions on the effectiveness of the Webex platform, used in Greece. It is one of the first studies that refer to the specific system, and uses primary data gathered by those directly involved, and more specifically the teachers. It can help reveal the advantages and disadvantages, as well as the problems encountered and help make suggestions with high educational impact, for the future. Besides, the COVID-19 pandemic has created a new reality which we all have to live with. Thus, it is an opportunity to study the role of IS in the COVID-19 pandemic, by referring to a major issue, that of education.

## 2 Research questions and objectives

The aim of the present study is to evaluate the use of the “Webex” online platform which was used during the quarantine – due to the COVID-19 pandemic – by the Greek public secondary education schools. The period under investigation is from March 2020 until June 2020, during the lockdown which was decided by the Greek government. Apart from Webex, other platforms, like e-class, were also used. Nevertheless, Webex was the platform used for the synchronous type of online education and it is more interesting to evaluate this platform, where interaction was in real time and both students and teachers needed to enter the platform at a prearranged time. As a result, deficiencies or inadequate interaction are easier to detect in the case of synchronous online education.

**Objectives of the Study** The specific objectives are.

• To assess the level of competency of teachers in using the new online environment.
• To determine whether teachers recognize the importance of online education.
• To evaluate the role of the state in supporting the transition to online learning.
• To measure the effectiveness of online education in achieving educational objectives.
• To identify the challenges faced by teachers and students during the transition.

According to the above-mentioned objectives, research questions were developed and are those listed below.

• Were teachers' and students' IT skills evaluated before and after the pandemic?
• Does the perceived utility of online education minimize obstacles and resistance to change?
• Was the state (Ministry of Education and Religious Affairs) supportive in terms of providing resources?
• What was the percentage of attendance?
• Were there any obstacles encountered?
• Were the educational objectives met?

**Fig. 1:**
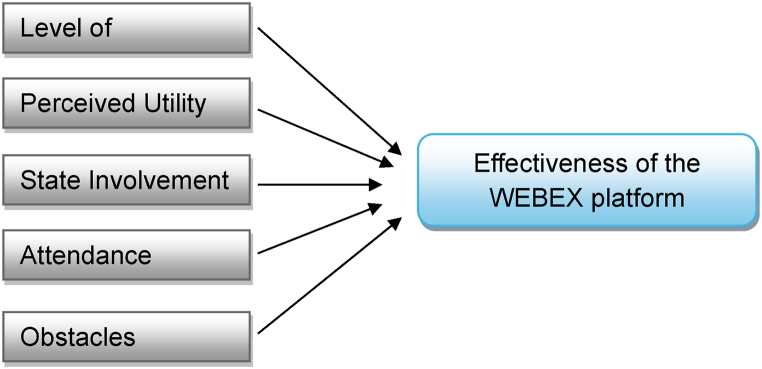
The research conceptual framework.

In order to better serve the research aim, the research conceptual framework was developed, in order to clarify the relationship among the primary research variables (see Fig. 1).

## 3 Literature review

The continuous advancement of Information Technology impacts all aspects of modern life. Digital technology is present in business, healthcare, communication, leisure activities, and virtually every private and public activity, so education could not be an exception. Online studying has been increasingly used over the last few decades and is widely believed by all stakeholders to represent the future of education at all levels [20]. The benefits of online classes and teaching have been widely acknowledged both before and after the COVID-19 pandemic.

Before the 2019 pandemic, online education was already praised for its accessibility, flexibility, and cost-effectiveness. It allowed students from diverse geographical locations to access quality education without the need for relocation and provided a flexible schedule to accommodate various personal and professional commitments. This flexibility was especially beneficial for working professionals, parents, and those with other responsibilities, helping to improve the balance between school and life and reducing stress levels [21,22].

Technological advancements have further improved the online learning experience by incorporating interactive platforms, virtual simulations, and multimedia resources, which have enhanced engagement and understanding. These tools not only made learning more effective but also supported lifelong learning by enabling individuals to continually update their skills and knowledge in response to changing job markets and personal interests [22,23]. Additionally, online education fostered global collaboration, allowing students and educators from diverse backgrounds to interact and share perspectives, thus promoting cross-cultural understanding and global awareness [24,25].

The COVID-19 pandemic accelerated the adoption of online education, highlighting its critical role in ensuring the continuity of learning amidst school closures affecting over a billion children worldwide [21]. The shift to e-learning platforms was marked by increased retention of information and reduced learning time, suggesting that the changes brought about by the pandemic might have lasting effects on the education sector [21]. The scalability of online education allowed it to accommodate large numbers of students, further demonstrating its potential to reach broader audiences compared to traditional classroom settings [22,23]. Despite challenges such as ensuring equitable access to technology and maintaining student engagement, the continuous improvements and innovations in online education suggest a promising future for this mode of learning [21,22].

### 3.1 Types of online learning

There are several types of online learning, which include the following [26].

• **Knowledgebase.** In this case, the education material is prepared and published online and general instruction is provided to students, without any further support.
• **Online support.** Online support type offers the potential of communication between the student and the tutor, in the form of a discussion board or a web-forum.
• **Asynchronous training.** During asynchronous training, the lessons are not provided in real-time, but students can enter the software and reach the educational material. In this case, the content is updated on a regular basis. Communication between students and tutors is realized through e-mails or messages on Social Media.
• **Synchronous training.** Unlike the case described above, synchronous training is realized in real time and the tutor and students can communicate with each other. In this case, the time to log-in is preset.
• **Hybrid training.** Hybrid training refers to the case where courses take place online but there is also physical interaction between the tutor and the student (e.g. through pre-set in-person meetings).

In Greece, asynchronous training, through e-class platform and synchronous training through Webex platform were applied. The COVID-19 pandemic has made isolation and physical distancing necessary, leading to the widespread implementation of e-learning across all levels of education. Information technology plays a crucial role in this new era, as it enables the integration of learning processes with the need for distance.

The new reality has both advantages and disadvantages. In several countries, including Greece, educational institutions, especially those of primary and secondary education, were not prepared to organize and conduct online courses. Governments issued the directive, but schools were expected to implement new teaching methods without prior training. Consequently, both teachers and students had to adapt to the new way of interacting in a short period of time. In Greece, academic research on distance learning during the pandemic is limited. Preliminary data indicates that three different distance learning schemes were implemented: asynchronous training, synchronous training, and Educational TV programs for primary school pupils [27]. Hence, IT became crucial in integrating learning processes with the need for distance education. However, several challenges emerged, including inadequate digital infrastructure, limited technology skills among educators and students, and the abrupt transition to online learning [1,5,6].

### 3.2 Global insights and challenges

Research, at a global level, mainly focuses on distant learning at university level. For instance, a study in Indonesia utilized a sample of students from the University of West Sulawesi and, by means of a questionnaire, found that online courses were both effective and inefficient [3]. They were effective in convincing students to participate in the courses, but inefficient in terms of cost. Furthermore, the research revealed that the vast majority of students (59 %) used the WhatsApp App application, followed by Zoom (8 %) and Facebook (3 %). This reveals that students prefer to use applications which can be loaded to their cell phones and are easy to use.

The Coronavirus epidemic has led researchers to the development of new term, that of Emergency Remote Teaching (ERT), which accurately describes the need for continuing the educational process in a different way [28].

Alvarez investigated the phenomenon of learning at a distance due to the COVID-19 epidemic, by discussing the experience of five learners in the Philippines, using the phenomenological approach [29]. The research findings revealed several issues reported by participants. Firstly, the lack of a reliable internet connection caused significant problems in the learning process. Secondly, financial constraints resulting from the epidemic hindered students from acquiring necessary equipment for online learning activities. Additionally, students indicated that they lacked the technological devices required to fully participate in lessons, highlighting that a mobile phone was insufficient. Finally, participants emphasized the importance of receiving emotional support as a crucial factor for their well-being. They expressed feelings of anxiety and fear and wished to feel safe and sound.

Almaiah et al. (2020) used the interview method in order to explore the challenges and factors influencing the e-learning system usage during the pandemic [7]. They used a sample of 30 students and 31 e-learning systems experts in two countries, Jordan and Saudi Arabia.

Another study conducted among university students in Fiji reported that poor internet access significantly hindered students' ability to participate fully in online classes. This issue was pervasive, affecting students' academic performance and engagement with learning activities [30].

In another research from the European University of Madrid it was emphasized that financial barriers substantially impacted students' access to educational resources during the pandemic. These constraints limited students' ability to acquire devices and internet services essential for participating in remote learning [31].

One other significant challenge of distance learning was the lack of suitable technological devices. More specifically, in the Philippines, students faced difficulties due to the insufficient capability of mobile phones to meet their educational needs. This was not an isolated issue, as other studies also found that students' learning experiences and academic performance were hindered by the absence of proper technological tools [32].

During the pandemic and remote learning, emotional support became crucial in helping students deal with stress and anxiety. For example, engineering students felt isolated and lacked support from instructors and peers, which affected their learning experience negatively. It was emphasized that emotional and psychological support is essential for student well-being and engagement in the shift to online learning [33].

The pandemic's psychological impact on students and teachers significantly influenced the online learning experience by causing stress, anxiety, and the need for social distancing, which affected overall teaching and learning environments [13]. Research has highlighted the significance of providing emotional support during online learning to help reduce these challenges [17]. The significance of maintaining social connections and providing emotional support was emphasized in various studies. These factors significantly contributed to students' overall satisfaction and academic success during the transition to distance education [32].

According to these research findings, the factors influencing mostly the usage of e-learning systems can be categorized as follows.

• Technological factors (physical equipment, software applications, operating systems)
• E-learning system quality factors (accessibility, availability, usability, personalization)
• Cultural factors (ICT literacy)
• Self-efficacy factors (IT skills)
• Trust factors (information privacy, system reliability)

### 3.3 Greece's experience with online education

As far as Greece is concerned, there have been no academic research results regarding the use of distance learning during the COVID-19 lockdown for secondary education. It's important to note that Greece faced a number of challenges in digitizing its society and economy. In 2019, Greece was ranked 26th out of 28 Member States in the EU's Digital Economy and Society Index (DESI), indicating outdated infrastructure and a lack of training among educators [1].

According to an article issued a month after the adoption of distance learning, students attended 32,585 daily digital classes, while educational TV had an average 35 % ratings [34]. Additionally, 643,871 students participated in daily synchronous online classes, with a 54 % increase each day [34]. Additionally, 95,692 teachers have set up their own digital classrooms [34]. After a month of operation, the "e-class" platform had over 1,000,000 students and 167,000 teachers registered [34]. The information provided above pertains to both primary and secondary education [34].

In addition, Andrianoupolitis (2020) found that the use of distance learning yielded more positive outcomes than anticipated [35]. In the case of universities, there were more positive results compared to primary and secondary education. Nevertheless, it appears that students have become even more familiar with IT, while it provided an opportunity to address important issues regarding the Greek educational system that had previously remained hidden. Some of the issues include the lack of IT infrastructure and insufficient training for teachers. Additionally, poor internet connections and high telecommunication costs are significant drawbacks. It has also become evident that the learning process is not as efficient as it is with physical interaction.

The analysis presented below reveals significant findings on the subject, reflecting the increasing importance of distance learning. This current research, based on the analysis of primary data, will contribute to the existing literature.

### 3.4 Research gaps

Despite the global shift to online education, there is limited research on the effectiveness of Webex in Greek secondary education during the pandemic. Existing studies have not fully explored the challenges and opportunities of the rapid transition to distance learning in a technologically unprepared setting like Greece [[11], [12], [13]].

More specifically, there is a significant gap in empirical data on the effectiveness of online learning platforms in Greek secondary education. Most research has focused on higher education, leaving secondary education underexplored [7,18]. In addition, inadequate IT training for teachers and students in Greece was a significant obstacle to effective online education and a crucial challenge for the government in continuing education [1,10]. Existing literature does not provide comprehensive insights into the IT competency levels required for successful online teaching [5,14]. Research on the role of state support and infrastructure in facilitating online education during the pandemic is limited. Hence, further investigation facilitates the assessment of the effectiveness of government initiatives and resource allocation [15,19].

The present study assesses the effectiveness of the Webex platform in Greece during the Covid-19 period. It constitutes one of the first studies referring to this specific system and uses primary data gathered directly from the teachers. It sheds light on the advantages and disadvantages, as well as the challenges encountered, and offers suggestions with a high educational impact for the future.

### 3.5 Direction of research and novelty

**Primary Data Collection:** This study aims to address research gaps by collecting primary data from secondary school teachers who used Webex during the quarantine period. By examining teachers' perspectives, this research provides valuable insights into IT competency, perceptions of online education, and state support. Even though this research was conducted during the Covid-19 pandemic and can be considered as Emergency Remote Teaching (ERT), the information gathered remains valid and useful. Despite the intense emotions surrounding ERT during the Covid-19 crisis, educators' feedback is valuable, as ERT also falls under educational policy-making. This period, despite being an emergency, motivates change, particularly in terms of embracing remote teaching and technology, which might not have been prioritized otherwise. According to policy-making theories, successful policy implementation often hinges on external factors that necessitate change.

According to Sabatier and Weible's Theories of the Policy Process (2014), external factors such as changes in political environments, economic conditions, and societal norms play pivotal roles in influencing policy implementation [36]. They discuss how frameworks like the Advocacy Coalition Framework (ACF) highlight the dynamic interactions between these external factors and policy outcomes. Similarly, Hill and Hupe's book, "Implementing Public Policy: Governance in Theory and Practice" (2009), emphasizes in Chapter 2 the significant impact of external influences on policy implementation [37]. Their book provides case studies and theoretical insights into how political, economic, and social factors shape the implementation process.

Furthermore, Howlett et al.'s book "Policy Styles and Policymaking: Exploring the Linkages" (2019) emphasizes in Chapter 1 how various policy styles adapt to external pressures and conditions [38]. The book, using case studies from different countries, explains the strategic adjustments policymakers need to make to align policies with changing external contexts, such as large-scale geohistorical developments [38]. These works highlight the influential role of external factors in shaping public policy implementation strategies and outcomes.

**Comprehensive Evaluation:** The current study assesses the impact of Webex on Greek secondary education, as an ERT policy, highlighting obstacles and psychological benefits. It examines teachers' proficiency in using the online environment, acknowledging the importance of online education, and evaluating the role of state support.

**Policy Implications**: The study's findings are meant to guide future educational policies and practices for politicians and decision-makers. This will help ensure that online teaching methods can be effectively integrated and supported. By addressing the identified challenges and providing actionable recommendations, the study contributes to the ongoing discussion on the future of education in a digitally connected world.

## 4 Methodology

The research will follow the specified research methodology to address the research questions. The research methodology provides the structure for conducting the research and analyzing and presenting the results. It is highly related to the researcher's view of the world [39]. First, research philosophy is determined. Research philosophy is “the system of beliefs and assumptions about the development of knowledge” [40]. For the present research, the suggested research philosophy is positivism. Positivism involves using real-life data to draw general conclusions by analyzing primary research results. In other words, the researcher will rely on real-life data to provide objective answers. The deductive approach in this study is characterized by the use of the mono-method, where only the survey method was employed. Through deduction, the researcher aims to draw general conclusions by studying a specific, representative sample [39]. Through deduction, a specific sample is analyzed, and results are generalized for the whole population. The inductive method was not used because it is more appropriate in the case of qualitative research [[41], [42], [43], [44], [45]].

In the presented study, the researcher opted for a deductive approach rather than an inductive one. The choice between these two approaches hinges on the nature of the research questions and the objectives of the study. A deductive approach begins with a general theory or hypothesis and then tests it through the collection and analysis of data [39]. This method is particularly suited to quantitative research, where the aim is to test specific hypotheses and generalize findings from a sample to a larger population. The deductive approach involves the following steps: theory development, hypothesis formulation, data collection, data analysis, and conclusion [46].

The **deductive approach** was utilized in this study for **several reasons.** Firstly, the research aimed to test specific hypotheses regarding the effectiveness of the Webex platform used by secondary education teachers during the pandemic. This necessitated the collection and analysis of quantitative data to determine whether the hypotheses are accurate [47]. The research aimed to apply findings from a sample of teachers to the broader population of secondary education teachers in Greece. Therefore, a deductive approach was chosen, as it enables drawing general conclusions from specific data.

Secondly, using structured, closed-type questions in the questionnaire enabled an objective analysis of the data, aligning with the deductive approach that emphasizes objectivity and the systematic testing of hypotheses [48]. Thus, employing closed-type questions, facilitate the researcher could categorize responses easily and reduce potential bias, making the data analysis more straightforward and reliable.

In contrast, an inductive approach involves collecting data and then developing a theory based on the analysis of that data. This approach is often used in qualitative research, where the goal is to explore phenomena and develop new theories rather than test existing ones [49]. The inductive approach typically involves data collection, identifying patterns and themes, and developing a theory based on observed patterns [47]. The study did not use the inductive approach because it relied on quantitative data collected through surveys, which is more compatible with the deductive approach. Additionally, the study aimed to test specific hypotheses about the usage and effectiveness of the Webex platform, rather than developing new theories from qualitative data [50]. Hence, the use of a deductive approach in this study is considered appropriate given the research objectives, the need for hypothesis testing, and the reliance on quantitative data. This approach made it easier to draw unbiased and applicable conclusions from the data gathered from a diverse group of high school teachers in Greece. The method's organized structure, along with the use of close-ended questions, guaranteed reliable findings that could be applied to a wider context.

The survey tool was chosen because it is a widely used method for gathering information on social issues, such as trends, attitudes and opinions [46]. More specifically, a questionnaire was created and sent to teachers who utilized the Webex platform during the final months of the Greek academic year. Through quantitative research, the obtained results are more objective and easier for the researcher to manage. In qualitative research, such as interviews, the researcher needs to interpret answers, which may introduce bias [51].

### 4.1 Description of participants & sampling technique

In order to complete the survey, individuals were chosen to answer the questionnaire. The characteristics of the participants, as well as the method of selection and the sample size, vary depending on the research. For instance, in qualitative research, the number of participants may be limited, and they are usually selected based on very strict and specific criteria [42].

On the other hand, quantitative research usually refers to large samples that are randomly selected. The research aim and objectives, as well as exogenous factors such as time availability, may affect the researcher's choice [39]. In this study, participants were chosen from the population of teachers, and since the research was quantitative, the following methodology was implemented [52].

**The sampling technique** used in this study is a form of non-probability sampling, specifically convenience sampling. Participants were chosen from the population of secondary education teachers who used the Webex platform during the COVID-19 pandemic. Recruitment was done through social media, where an invitation message was posted and shared among secondary education teachers. The characteristics required for participation included being a secondary education teacher, being employed in a school in Greece, and having used the Webex platform for online teaching during the pandemic. A total of 112 teachers responded to the questionnaire, forming the final sample used for the analysis.

This sampling method is considered to be non-parametric because it does not involve random selection from the entire population of Greek secondary education teachers. Instead, it relies on the availability and willingness of teachers to participate, which is characteristic of non-probability sampling [39]. Convenience sampling is evident due to the recruitment method (social media) and the lack of a systematic approach to ensure an equal chance of selection for every teacher in the population. The use of social media to reach participants and reliance on volunteers classifies it as a convenience sampling technique [42].

Given the urgency and context of the pandemic, convenience sampling was practical for quickly gathering data from a specific group of teachers who had recent experience with the Webex platform [48]. The lack of randomization, as there was no use of random selection methods such as random number generation or systematic sampling lists, is typical in parametric sampling techniques. Additionally, the sample includes teachers who voluntarily participated. This may introduce bias, as those more comfortable with technology or with stronger opinions about the online platform may be overrepresented [52]. However, it should be noted that the emergence of smart devices, such as smartphones and tablets, has significantly simplified the use of social media, even for people with no technological skills.

The sampling technique used in this study is non-parametric, specifically convenience sampling. This is determined based on the method of recruitment (social media), the reliance on voluntary participation, and the lack of random selection, which are characteristic of non-probability sampling methods [51]. This approach was practical given the study's context and objectives, despite potential biases associated with convenience sampling.

The survey included teachers working in secondary education across various regions who utilized the Webex platform. In order to gather the necessary data, a sampling method frequently used in quantitative research was employed. Specifically, participants serving as the research sample were randomly chosen from the pool of secondary education teachers. This random selection is crucial for quantitative research, as it necessitates statistical analysis [40]. Consequently, the research population consists of secondary education teachers from public schools in Greece. These participants represent a sample percentage of this population [53].

The survey participants needed to possess the following characteristics in order to take part in the survey.

✓ They needed to be secondary education teachers.
✓ They needed to work in a school within the country.
✓ They needed to have used the Webex platform.

No other restrictions or special requests were made.

Participants were recruited through social media. The procedure involved the researchers posting a message inviting secondary education teachers to complete a questionnaire about the online platform used in distance synchronous education.The message is described below (Detailed and descriptive data collection procedure). Even though more questionnaires were collected, the final sample, which includes useable questionnaires, consists of 112 teachers working in various regions of Greece. Only questionnaires with complete answers to all questions were considered useable [54].

Ethical issues as well as personal data protection issues (GDPR) were taken into consideration for all participants. When individuals are involved in a research, some issues concerning participants’ dignity and safety need to be considered [55].

The primary concern is voluntary participation. It is crucial that individuals who take part in the research do so of their own volition. This was ensured by obtaining participants’ consent at the end of the information letter, which was provided at the start of the questionnaire. The complete letter can be found in the appendix.

Confidentiality and anonymity are sensitive issues that researchers need to consider carefully. Participants were informed about how their answers would be handled, and no personal data was collected. To protect data, questionnaires were destroyed after they were analyzed.

The preceding factors were all taken into consideration. The informative text included all necessary details to ensure the safety of participants, protect their anonymity, allow them to withdraw from the survey, and confirm that they participated with their consent. The informative letter and questionnaire are provided in appendix. A comprehensive analysis of the respondents' descriptions will follow.

### 4.2 Description of treatment and data collection tools

In the previous section, we mentioned that we used a questionnaire as our data collection tool because it is widely used and easy to analyze. Additionally, it is a cost-effective and efficient method as many people can respond within a short time period. By using the questionnaire, we were able to reach a significant number of participants [39], which in our analysis is essential, due to the aim of the research, which focuses on users’ satisfaction and pedagogical aims achievement.

The use of closed-type questions allowed for better categorization and analysis of the results. Closed-type questions restrict participants from elaborating on their ideas and thoughts, instead requiring them to select from predetermined answers. Additionally, closed-type questions are easier for the researcher to analyze [39]. The other option would be "open-ended questions." With open-ended questions, participants can freely express their opinions. However, it's more challenging for the research to categorize the answers and prevent bias. Additionally, open-ended questions are suitable for qualitative research [40].

There are several types of “closed type” questions. For the purposes of the present study, the following types were used. The types selected are those that were found to be more suitable for the research, so as to answer the research questions [56].

- Dichotomous questions, which are answered with “yes” or “no.”
- Nominal type questions, where participants are invited to select among several statements.
- Likert scale questions, where participants need to express their preference according to a scale. In the present case, the 5-point Likert Scale was used and participants had to state whether they “totally disagree/disagree/neither agree nor disagree/agree/totally agree” with a given statement.

**Examples**.

➢ Dichotomous question: “Did you have the potential to contact a “help desk” developed by the ministry of education, in case of trouble?
  ❒ 1. Yes
  ❒ 2. No”
➢ Likert Scale question: “The ministry of education and religious affairs provided several options concerning alternative platforms.” (Please tick on the level of agreement withthe above-mentioned statement)
➢ ❒ 1. Totally disagree
➢ ❒ 2. Disagree
➢ ❒ 3. Neither agree, nor disagree
➢ ❒ 4. Agree
➢ ❒ 5. Totally agree”

Nominal type question: “The percentage of attendance during the period was.

➢ ❒ 1. Increased day-by-day
➢ ❒ 2. Decreased day-by-day
➢ ❒ 3. Remained the same
➢ ❒ 4. Fluctuated

The questionnaire was created after conducting a thorough literature review and developing a preliminary research questionnaire. This preliminary questionnaire was then refined and finalized. Additionally, the questionnaire was written in a clear and simple manner to make it user-friendly and encourage participants to complete it [57]. Questions were divided in groups. First, demographical questions were asked, and then questions referring to the following categories followed.

➢ Questions referring to the level of competency.
➢ Questions referring to perceived utility.
➢ Questions referring to the involvement of the official state.
➢ Questions referring to attendance levels.
➢ Questions referring to obstacles encountered.
➢ Questions referring to the educational goal's achievement (transition of class to e-class content, e-class activities at home).
➢ Questions referring to the effectiveness of the platform.

The questionnaire was tested in advance. It was distributed to five individuals to identify duplicate questions, logical errors, spelling or grammatical errors, and irrelevant or difficult-to-understand questions [46].

### 4.3 Detailed and descriptive data collection procedure

Data collection procedures are crucial as they directly impact the credibility of research findings. Credibility consists of two dimensions: reliability and validity [58]. Reliability encompasses both the data collection procedure and the data analysis process. When it comes to data collection, the researcher must be diligent in addressing threats to reliability. One such threat is "participant error," which highlights the significance of ensuring that participants are in an appropriate physical and psychological state to answer the questions accurately. In this study, participants were given the freedom to complete the questionnaire at their convenience. The survey took place during the summer when teachers have more free time, but this timing could lead to bias. Participants might feel pressured to give responses that align with what their supervisors or the education ministry want to hear. This type of bias can be difficult to detect, so the researcher needs to carefully analyze the data to identify any potential biases. To address this issue, the study used similar questions in different parts of the questionnaire and also ensured participant anonymity.

Validity is also related to credibility [59]. Validity is affected by several factors. It's essential to keep in mind that participants' opinions may not be entirely objective if they are asked to give their feedback right after being affected by a certain situation. For example, in the case of online education, students faced various obstacles that affected the effectiveness of the process. However, during the research, they may overlook these obstacles and only recall the positive impact on students' psychology. It's important to note that there may have been less immediate psychological stress while filling out the questionnaires in the summer compared to during the peak of the coronavirus crisis (spring semester).

The analysis considered the previously mentioned factors and then proceeded to data collection. Data collection was carried out according to the following procedure: Teachers were invited to participate through social media. Specifically, the researcher developed a brief message and posted it on social media, inviting their "friends" to share the message with their own friends. The message was as follows:

“We kindly invite you to take part in an academic research referring to the effectiveness of online education during the lockdown caused by the COVID-19 pandemic. We value your opinion and hope the present study becomes a reference point for the specific subject, by revealing interest aspects of it. The study is independent, and your anonymity will be preserved. It will take only a few minutes to answer the enclosed questionnaire, while your participation will be valuable. Thank you in advance!”

The message was sent out via Facebook, Twitter, and email to secondary education teachers. It was sent every day for ten days, from July 16th to July 25th, 2020, at three different times. At the end of the ten days, 128 questionnaires were filled out. The questionnaires went through three stages of processing: a validity check, a descriptive check/process in Microsoft Excel, and a final statistical analysis/process in SPSS. Specifically, each questionnaire was carefully reviewed for mistakes or omissions, such as selecting two answers instead of one.

After completing the process, we had 112 useable questionnaires. We then encoded and processed the answers using Microsoft Excel. The analysis proceeded in two steps: descriptive analysis and statistical analysis. The latter aimed to identify relationships among the variables based on the conceptual framework. Additionally, we calculated the mean scores of answers to questions in each group using Excel. These steps were followed to eliminate errors, safeguard the process, and ensure data validity and protection. Subsequently, the data was entered into SPSS for both descriptive and statistical analysis.

## 5 Data analysis and presentation of results of findings

**Fig. 2:**
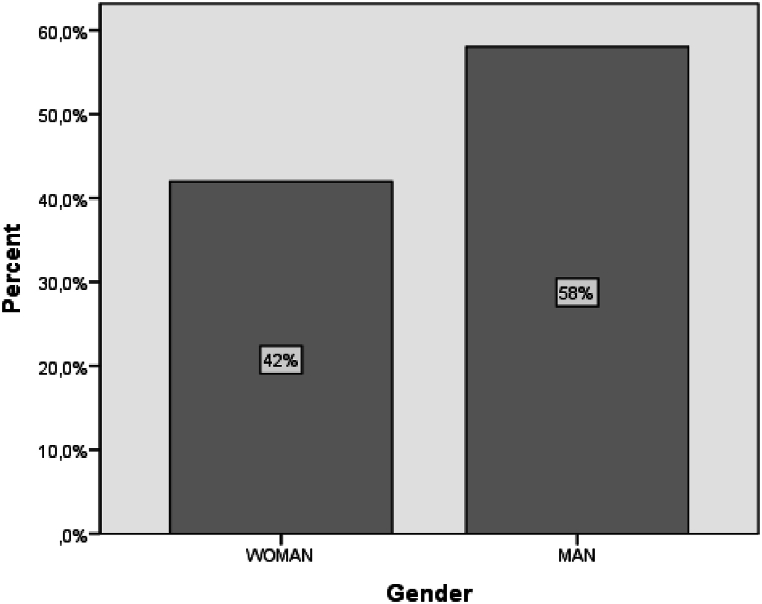
The gender of the sample.

**Fig. 3:**
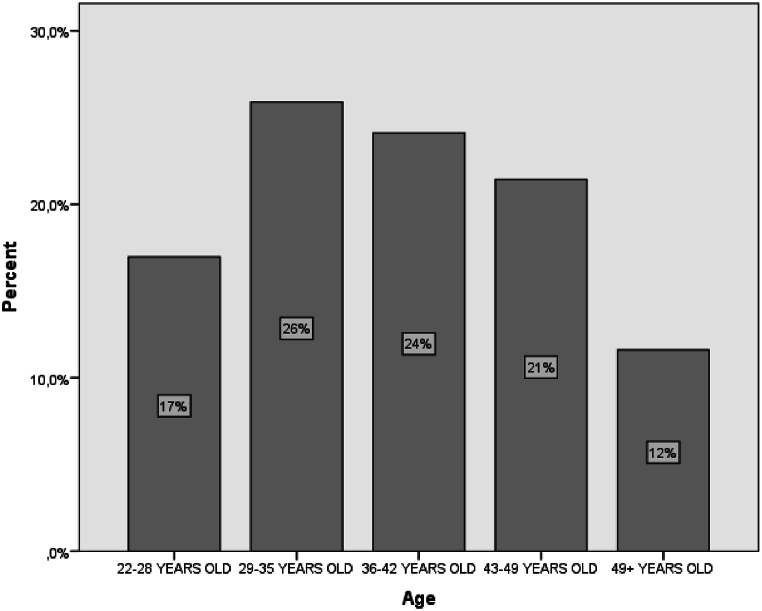
The age of the sample.

In order to achieve the research aims and objectives, the answers to the research questions are presented below through data analysis. Firstly, descriptive analysis is presented, and the results are grouped based on the structure of the questionnaire. Participants' demographics are presented first, followed by the answers to each group of questions based on the research question. Statistical analysis is then used to reveal the factors that determine effective online education (Fig. 2, Fig. 3).

a **Questions Referring to Demographics**

**Fig. 4:**
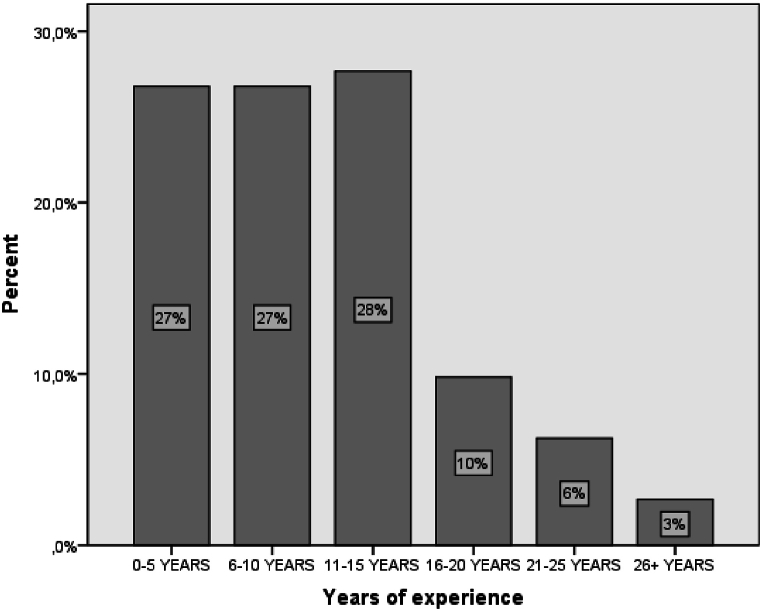
The sample's years of experience.

In the study, 58 % of the participants were men, and 42 % were women. In terms of age distribution, 26 % were between 29 and 35 years old, 24 % were between 36 and 42 years old, 21 % were between 22 and 28 years old, and only 12 % were over 49 years old. These findings indicate that the majority of participants were under 42 years old, which could potentially impact the results, as younger individuals generally have more proficiency with new technologies compared to older individuals (Fig. 4).

**Fig. 5:**
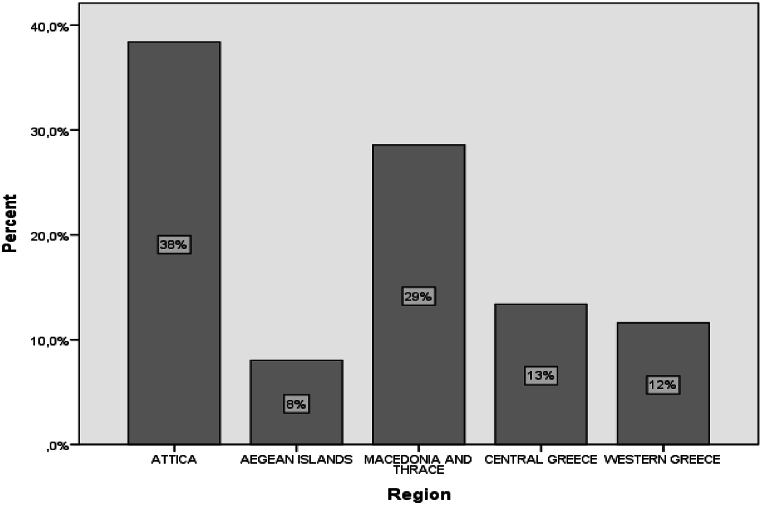
The sample region of employment.

The following demographic data pertains to years of experience. The majority of participants had less than 15 years of experience, with 28 % having 11–15 years, 27 % having 6–10 years, and another 27 % having 0–5 years. 10 % of teachers had 16–20 years of experience, 6 % had 21–25 years, and only 3 % had more than 26 years of experience (Fig. 5).

The geographical distribution of the teachers' workplaces was documented. The majority of participants were employed in Attica (38 %) and Macedonia-Thrace (29 %). This is understandable, as these regions encompass the major Greek cities of Athens and Thessaloniki, which have high population densities. 13 % of participants work in Central Greece, 12 % in Western Greece, and 8 % on the Aegean Islands. The research is comprehensive, as the sample includes teachers from all regions of the country.

b **Questions on the Level of Competency**

Questions regarding teacher competency in responding to IT skills were developed to assess their proficiency in the new environment. The results are presented in the following tables (Table 1).

**Table 1:** Answers to the question: Are you qualified by an official institution on IT subjects?

Are you qualified by an official institution on IT subjects?					
		Frequency	Percent	Valid Percent	Cumulative Percent
Valid	YES	55	49,1	49,1	49,1
	NO	57	50,9	50,9	100,0
	Total	112	100,0	100,0	

According to the results, approximately half of the participants were formally qualified in IT subjects before using the “Webex” platform. This percentage is unsatisfactory, considering that technology has now become integrated into all aspects of private and professional life (Table 2).

**Table 2:** Answers to the question: Were you asked to participate in an evaluation test concerning your competence, before using "Webex"?

Were you asked to participate in an evaluation test concerning your competence, before using "Webex"?					
		Frequency	Percent	Valid Percent	Cumulative Percent
Valid	YES	18	16,1	16,1	16,1
	NO	94	83,9	83,9	100,0
	Total	112	100,0	100,0	

The majority of teachers (84 %) were not asked to take part in an evaluation test of their competence, indicating that their competency was considered a given. However, this contradicts the previous question, which suggested otherwise (Table 3).

**Table 3:** Answers to the questions: "I am competent in new technologies and especially internet usage".

"I am competent in new technologies and especially internet usage"					
		Frequency	Percent	Valid Percent	Cumulative Percent
Valid	TOTALLY DISAGREE	3	2,7	2,7	2,7
	DISAGREE	23	20,5	20,5	23,2
	NEITHER AGREE, NOR DISAGREE	35	31,3	31,3	54,5
	AGREE	47	42,0	42,0	96,4
	TOTALLY AGREE	4	3,6	3,6	100,0
	Total	112	100,0	100,0	

As far as participants’ competency in new technologies is concerned, more than one-third of them are unsure of their ability to respond to new technology needs. Furthermore, even though 42 % of them agree that they are competent, 23 % disagree, and 3 % totally disagree. The cumulative percentage is high (26 %) and needs to be considered (Table 4).

**Table 4:** Answers to the question: "I found it easy to work through the "Webex" platform".

"I found it easy to work through the "Webex" platform"					
		Frequency	Percent	Valid Percent	Cumulative Percent
Valid	TOTALLY DISAGREE	4	3,6	3,6	3,6
	DISAGREE	17	15,2	15,2	18,8
	NEITHER AGREE, NOR DISAGREE	36	32,1	32,1	50,9
	AGREE	48	42,9	42,9	93,8
	TOTALLY AGREE	7	6,3	6,3	100,0
	Total	112	100,0	100,0	

The responses to the question mentioned earlier showed that half of the participants found it easy to work through the "Webex" platform, while 32 % of them found it neither easy nor difficult, and 19 % found it difficult. These results also indicate that a significant percentage of teachers are not well-prepared to effectively use online platforms (Table 5).

**Table 5:** Answers to the question: "I was even able to help students with the problems they encountered concerning the usage of the platform".

"I was even able to help students with the problems they encountered concerning the usage of the platform"					
		Frequency	Percent	Valid Percent	Cumulative Percent
Valid	TOTALLY DISAGREE	10	8,9	8,9	8,9
	DISAGREE	30	26,8	26,8	35,7
	NEITHER AGREE, NOR DISAGREE	34	30,4	30,4	66,1
	AGREE	37	33,0	33,0	99,1
	TOTALLY AGREE	1	,9	,9	100,0
	Total	112	100,0	100,0	

In the final question, participants were asked whether teachers could assist students with any problems they faced. 34 % of the respondents had a positive view, while 30 % were neutral, and 36 % held a negative opinion. Overall, the responses regarding teachers' competence in IT indicate that at least half of the participants require further training, as they are not sufficiently qualified and lack confidence in using the "Webex" online platform.

c **Questions on Perceived Utility**

The next set of questions pertains to perceived usefulness. Answers to each question are provided below (Table 6).

**Table 6:** Answers to the group of questions referring to perceived utility.

"The online platform was a necessity, so as to continue academic lessons"					
		Frequency	Percent	Valid Percent	Cumulative Percent
Valid	TOTALLY DISAGREE	1	,9	,9	,9
	DISAGREE	7	6,3	6,3	7,1
	NEITHER AGREE, NOR DISAGREE	23	20,5	20,5	27,7
	AGREE	65	58,0	58,0	85,7
	TOTALLY AGREE	16	14,3	14,3	100,0
	Total	112	100,0	100,0	
**"I am willing to continue using the platform, no matter the obstacles, since it helps students stay in touch with the educational process"**					
		Frequency	Percent	Valid Percent	Cumulative Percent

Valid	DISAGREE	10	8,9	8,9	8,9
	NEITHER AGREE, NOR DISAGREE	11	9,8	9,8	18,8
	AGREE	59	52,7	52,7	71,4
	TOTALLY AGREE	32	28,6	28,6	100,0
	Total	112	100,0	100,0	
**"I am willing to participate in seminars in order to become more competent because I believe in the utility of distant learning"**					
		Frequency	Percent	Valid Percent	Cumulative Percent

Valid	TOTALLY DISAGREE	1	,9	,9	,9
	DISAGREE	8	7,1	7,1	8,0
	NEITHER AGREE, NOR DISAGREE	15	13,4	13,4	21,4
	AGREE	83	74,1	74,1	95,5
	TOTALLY AGREE	5	4,5	4,5	100,0
	Total	112	100,0	100,0	

The results of the survey indicate that the majority of participants recognize the online platform as essential. 83 % of them are willing to continue using the platform, despite any obstacles, because they believe it helps students stay connected to the educational process. It's also noteworthy that 79 % of teachers are willing to attend seminars to improve their skills, showing their sense of duty.

d **Questions on the Involvement of the State**

The state plays a crucial role in implementing and ensuring the success of changes in the educational system. Therefore, questions about state support and involvement have been developed, and the answers to this group of questions follow (Table 7).

**Table 7:** Answers to the question: "Did you receive official training on the usage of the platform?"

"Did you receive official training on the usage of the platform?"					
		Frequency	Percent	Valid Percent	Cumulative Percent
Valid	NO	112	100,0	100,0	100,0

Answers to the first question in this group are indicative of the situation. All participants answered that no official training was conducted prior to using the platform. In fact, the Greek Ministry of Education and Religious Affairs provided guidance through its official website, but this was the only way teachers could receive some information on how the platform worked (Table 8).

**Table 8:** Answers to the question: "The state provided the necessary infrastructure".

"The state provided the necessary infrastructure"					
		Frequency	Percent	Valid Percent	Cumulative Percent
Valid	TOTALLY DISAGREE	9	8,0	8,0	8,0
	DISAGREE	75	67,0	67,0	75,0
	NEITHER AGREE, NOR DISAGREE	17	15,2	15,2	90,2
	AGREE	11	9,8	9,8	100,0
	Total	112	100,0	100,0	

As for the infrastructure, teachers had to have their own computers or use those provided at school. In fact, 75 % of participants answered that no infrastructure was provided, 15 % were neutral, and only 10 % answered positively (Table 9).

**Table 9:** Answers to the question: "The ministry of education and religious affairs provided the necessary guidance for the usage of the platform".

"The ministry of education and religious affairs provided the necessary guidance for the usage of the platform"					
		Frequency	Percent	Valid Percent	Cumulative Percent
Valid	DISAGREE	41	36,6	36,6	36,6
	NEITHER AGREE, NOR DISAGREE	34	30,4	30,4	67,0
	AGREE	37	33,0	33,0	100,0
	Total	112	100,0	100,0	

According to the responses, the Ministry provided guidance through its website. However, 37 % of the participants found the guidance inadequate, while 30 % were unsure about its adequacy. Only 33 % of the participants found the guidance to be adequate. This could be attributed to a significant number of participants lacking IT competency (Table 10).

**Table 10:** Answers to the question: "The ministry of education and religious affairs provided several options concerning alternative platforms".

"The ministry of education and religious affairs provided several options concerning alternative platforms"					
		Frequency	Percent	Valid Percent	Cumulative Percent
Valid	DISAGREE	100	89,3	89,3	89,3
	AGREE	12	10,7	10,7	100,0
	Total	112	100,0	100,0	

The final question in the survey is whether teachers had the choice to use multiple synchronous teaching platforms. 89 % of the participants answered "no." The Ministry had recommended using only the "Webex" platform for synchronous teaching and the "e-class" platform for asynchronous education. However, some teachers opted to use other platforms such as "Skype" because they allowed for increased interaction. This decision, however, led to confusion among parents, as they had to log in to different platforms.

e **Questions on the Levels of Attendance**

Below, participants’ responses to the questions regarding attendance levels are provided to reveal the extent to which students engaged with the new teaching method (Table 11).

**Table 11:** Answers to the question: "Which was the percentage of attendance after the first week?"

"Which was the percentage of attendance after the first week?"					
		Frequency	Percent	Valid Percent	Cumulative Percent
Valid	0–20 %	13	11,6	11,6	11,6
	20%–40 %	36	32,1	32,1	43,8
	40%–60 %	45	40,2	40,2	83,9
	60%–80 %	13	11,6	11,6	95,5
	80%–100 %	5	4,5	4,5	100,0
	Total	112	100,0	100,0	

Based on responses to the initial question, attendance varied between 20 % and 60 % after the first week. Specifically, 40 % of teachers reported that 40%–60 % of the total number of students attended the lessons through the "Webex" platform. 32 % stated that attendance was between 20% and 40 %, while only 5 % of the teachers reported that the attendance was more than 80 % (Table 12).

**Table 12:** Answers to the questions: "The percentage of attendance during the period was:"

"The percentage of attendance during the period was:"					
		Frequency	Percent	Valid Percent	Cumulative Percent
Valid	increasing day-by-day	69	61,6	61,6	61,6
	decreasing day-by-day	2	1,8	1,8	63,4
	remaining the same	21	18,8	18,8	82,1
	fluctuating	20	17,9	17,9	100,0
	Total	112	100,0	100,0	

Even though the attendance percentage after the first week is not encouraging, it is important to note that in the majority of cases (62 %), this percentage increased day by day (Table 13).

**Table 13:** Answers to the question: "I am pleased with the percentage of attendance".

"I am pleased with the percentage of attendance"					
		Frequency	Percent	Valid Percent	Cumulative Percent
Valid	TOTALLY DISAGREE	6	5,4	5,4	5,4
	DISAGREE	80	71,4	71,4	76,8
	NEITHER AGREE, NOR DISAGREE	15	13,4	13,4	90,2
	AGREE	9	8,0	8,0	98,2
	TOTALLY AGREE	2	1,8	1,8	100,0
	Total	112	100,0	100,0	

The fact that the percentage of participants on the "Webex" platform is increasing daily is not satisfactory for teachers. The majority of them (77 %) are not pleased with the attendance rate. This is reasonable since teachers want all students to be able and willing to participate in their lessons.

f **Questions on the Obstacles Encountered**

Questions about encountered obstacles are important as they can guide further improvement. Answers to these questions are provided below (Table 14).

**Table 14:** Answers to questions referring to the obstacles encountered.

“Students could not attend the online courses due to financial constraints”					
		Frequency	Percent	Valid Percent	Cumulative Percent
Valid	DISAGREE	47	42,0	42,0	42,0
	NEITHER AGREE, NOR DISAGREE	38	33,9	33,9	75,9
	AGREE	27	24,1	24,1	100,0
	Total	112	100,0	100,0	
**"There were not connectivity issues during the on line courses"**					
		Frequency	Percent	Valid Percent	Cumulative Percent

Valid	TOTALLY DISAGREE	20	17,9	17,9	17,9
	DISAGREE	65	58,0	58,0	75,9
	NEITHER AGREE, NOR DISAGREE	18	16,1	16,1	92,0
	AGREE	9	8,0	8,0	100,0
	Total	112	100,0	100,0	
**"There were not technological issues that needed troubleshooting during the on-line courses"**					
		Frequency	Percent	Valid Percent	Cumulative Percent

Valid	DISAGREE	25	22,3	22,3	22,3
	NEITHER AGREE, NOR DISAGREE	16	14,3	14,3	36,6
	AGREE	71	63,4	63,4	100,0
	Total	112	100,0	100,0	
**"Students were on time and entered the platform before and not during the online lesson"**					
		Frequency	Percent	Valid Percent	Cumulative Percent

Valid	DISAGREE	34	30,4	30,4	30,4
	NEITHER AGREE, NOR DISAGREE	13	11,6	11,6	42,0
	AGREE	64	57,1	57,1	99,1
	TOTALLY AGREE	1	,9	,9	100,0
	Total	112	100,0	100,0	

According to participants’ answers, the following can be said on the obstacles encountered.

• According to 24 % of participants, students' financial problems were an obstacle, while 34 % were uncertain.
• During the online courses, 76 % of participants reported experiencing connectivity issues.
• 22 % of participants reported encountering IT issues during the courses. The data indicates that connectivity issues were the most significant obstacle, and this should be taken into consideration by the authorities. Further investigation is required to determine whether connectivity issues are related to different platforms or network overload.

These results indicate that the primary obstacle was connectivity issues, which the state must address. Further investigation is required to determine if connectivity issues were caused by different platforms or backbone network overload.

g **Questions on the Educational Goals**

The questions about educational goals were created to determine if students are able to receive the same level of education as before. Additionally, they aimed to uncover any differences between the new and previous methods of training. The responses to each question are provided below (Table 15).

**Table 15:** Answers to the question: "The number of activities and assignments are appropriate (compared to the previous status), so the workload is reasonable".

"The number of activities and assignments are appropriate (compared to the previous status), so the workload is reasonable"					
		Frequency	Percent	Valid Percent	Cumulative Percent
Valid	TOTALLY DISAGREE	2	1,8	1,8	1,8
	DISAGREE	24	21,4	21,4	23,2
	NEITHER AGREE, NOR DISAGREE	24	21,4	21,4	44,6
	AGREE	60	53,6	53,6	98,2
	TOTALLY AGREE	2	1,8	1,8	100,0
	Total	112	100,0	100,0	

According to the responses to the first question, the majority of teachers (56 %) find the current workload reasonable compared to the previous status. However, it's also important to note that a significant percentage (26 %) disagree (Table 16).

**Table 16:** Answers to the question: "Selected readings and resources reflect and fit the subject and course learning outcomes".

"Selected readings and resources reflect and fit the subject and course learning outcomes"					
		Frequency	Percent	Valid Percent	Cumulative Percent
Valid	TOTALLY DISAGREE	2	1,8	1,8	1,8
	DISAGREE	18	16,1	16,1	17,9
	NEITHER AGREE, NOR DISAGREE	60	53,6	53,6	71,4
	AGREE	31	27,7	27,7	99,1
	TOTALLY AGREE	1	,9	,9	100,0
	Total	112	100,0	100,0	

Participants were asked whether selective reading and resources align with the subject and course learning outcomes. The results showed that 53 % of participants were unsure, 18 % disagreed, and 29 % agreed. Based on this feedback, it is evident that selective reading and resources require further evaluation before being recommended to the students (Table 17).

**Table 17:** Answers to the question: "Students have the opportunity for interaction with the content".

"Students have the opportunity for interaction with the content"					
		Frequency	Percent	Valid Percent	Cumulative Percent
Valid	TOTALLY DISAGREE	32	28,6	28,6	28,6
	DISAGREE	78	69,6	69,6	98,2
	NEITHER AGREE, NOR DISAGREE	2	1,8	1,8	100,0
	Total	112	100,0	100,0	

A significant benefit of live classes is the opportunity for interaction, which is lacking in online courses, according to participants' feedback. Teachers have noted that students do not have the chance to engage with the content, which is a drawback of the platform. This limitation, although it may be attributed to the platform, is a general disadvantage that applies to all distance learning platforms and tools (Table 18).

**Table 18:** Answers to the question: "Students' involvement and activities are adequate in order for them to acquire the knowledge needed".

"Students' involvement and activities are adequate in order for them to acquire the knowledge needed"					
		Frequency	Percent	Valid Percent	Cumulative Percent
Valid	TOTALLY DISAGREE	12	10,7	10,7	10,7
	DISAGREE	50	44,6	44,6	55,4
	NEITHER AGREE, NOR DISAGREE	30	26,8	26,8	82,1
	AGREE	20	17,9	17,9	100,0
	Total	112	100,0	100,0	

The final question is a general one and indicates that teachers are dissatisfied with how students are engaged in the process, as well as with the provided activities. Specifically, only 18 % of participants believe that students' involvement and activities are sufficient, while 55 % of them disagree and 27 % were neutral. In summary, it appears that teachers only agree on the amount of workload and whether it is reasonable. The responses to other questions indicate that improvements are necessary in terms of interaction, students' involvement, and activities.

h **Questions on the Effectiveness of the Platform**

The last set of questions pertains to the overall effectiveness of the platform. Responses are listed in the table below (Table 19).

**Table 19:** Answers to the questions on the effectiveness of the platform.

"I think the whole project was beneficial for the students, in terms of learning"					
		Frequency	Percent	Valid Percent	Cumulative Percent
Valid	TOTALLY DISAGREE	4	3,6	3,6	3,6
	DISAGREE	61	54,5	54,5	58,0
	NEITHER AGREE, NOR DISAGREE	16	14,3	14,3	72,3
	AGREE	30	26,8	26,8	99,1
	TOTALLY AGREE	1	,9	,9	100,0
	Total	112	100,0	100,0	
**"I think the project was beneficial for students, in terms of emotional support"**					
		Frequency	Percent	Valid Percent	Cumulative Percent

Valid	DISAGREE	13	11,6	11,6	11,6
	NEITHER AGREE, NOR DISAGREE	22	19,6	19,6	31,3
	AGREE	62	55,4	55,4	86,6
	TOTALLY AGREE	15	13,4	13,4	100,0
	Total	112	100,0	100,0	
**"I am willing to continue with this type of learning**					
		Frequency	Percent	Valid Percent	Cumulative Percent

Valid	DISAGREE	52	46,4	46,4	46,4
	NEITHER AGREE, NOR DISAGREE	15	13,4	13,4	59,8
	AGREE	44	39,3	39,3	99,1
	TOTALLY AGREE	1	,9	,9	100,0
	Total	112	100,0	100,0	

According to the teachers' responses, it is interesting that even though the majority of them (58 %) did not find the courses beneficial in terms of learning, they found them beneficial in terms of emotional support (69 %). This is very important and indicative of the emotional impact of the pandemic on students. Regarding whether teachers are willing to continue with this type of learning, 46 % of them disagreed, 40 % agreed, and 14 % were neutral. These results should alert the authorities and prompt them to improve the aspects of online education.

The purpose of this study is to assess the effectiveness of the "Webex" platform. To do this, a conceptual framework was developed that includes the variables related to effectiveness. Several sets of questions were created, each addressing a specific variable. After presenting and discussing the results, correlation analysis was conducted to explore the relationships between the variables and their correlation with the platform's effectiveness. Mean scores for the questions in each group were calculated. Non-Likert scale questions were excluded in order to prevent them from influencing the results. The descriptive statistics for the grouped data are provided below (Table 20, Table 21).

**Table 20:** Descriptive statistics of grouped variables.

Descriptive Statistics					
	N	Minimum	Maximum	Mean	Std. Deviation
LEVEL OF COMPETENCY	112	1,33	4,33	3,1551	,70292
PERCEIVED UTILITY	112	1,67	4,67	3,8451	,67667
STATE INVOLVEMENT	112	2,00	4,00	2,5893	,53362
ATTENDANCE	112	1,00	5,00	2,4732	,82155
OBSTACLES	112	2,00	4,00	3,0045	,45518
EDUCATIONAL GOALS	112	1,50	3,50	2,6674	,45814
EFFECTIVENESS	112	2,00	4,67	3,1039	,67563
Valid N (listwise)	112

**Table 21:** Correlations among grouping variables.

Correlations								
		LEVEL OF COMPETENCY	PERCEIVED UTILITY	STATE INVOLVEMENT	ATTENDANCE	OBSTACLES	EDUCATIONAL GOALS	EFFECTIVENESS
LEVEL OF COMPETENCY	Pearson Correlation	1	,411^a^	,343^a^	-,139	,143	-,142	-,029
	Sig. (2-tailed)		,000	,000	,145	,132	,136	,765
PERCEIVED UTILITY	Pearson Correlation	,411^a^	1	,271^a^	-,105	,062	-,039	-,016
	Sig. (2-tailed)	,000		,004	,271	,518	,680	,870
STATE INVOLVEMENT	Pearson Correlation	,343^a^	,271^a^	1	-,056	,124	,007	,040
	Sig. (2-tailed)	,000	,004		,557	,194	,938	,672
ATTENDANCE	Pearson Correlation	-,139	-,105	-,056	1	,178	,009	-,238^b^
	Sig. (2-tailed)	,145	,271	,557		,060	,925	,011
OBSTACLES	Pearson Correlation	,143	,062	,124	,178	1	-,120	-,217^b^
	Sig. (2-tailed)	,132	,518	,194	,060		,209	,022
EDUCATIONAL GOALS	Pearson Correlation	-,142	-,039	,007	,009	-,120	1	,453^a^
	Sig. (2-tailed)	,136	,680	,938	,925	,209		,000
EFFECTIVENESS	Pearson Correlation	-,029	-,016	,040	-,238^b^	-,217^b^	,453^a^	1
	Sig. (2-tailed)	,765	,870	,672	,011	,022	,000	

a Correlation is significant at the 0.01 level (2-tailed).

b Correlation is significant at the 0.05 level (2-tailed).

According to the table, the variables that show statistically significant correlation (up to 5 %) are as follows.

• The level of competency is correlated with perceived utility (r = 0.45, p < 0.05) and state involvement (r = 0.38, p < 0.05).
• Perceived utility is correlated with state involvement (r = 0.42, p < 0.05).
• Effectiveness is statistically correlated with attendance (r = −0.30, p < 0.05), obstacles (r = −0.47, p < 0.05), and educational goals (r = 0.52, p < 0.05).

The results show that the platform's effectiveness is statistically significantly correlated with only three out of the six variables in the research conceptual framework. It's also noteworthy that the platform's effectiveness is negatively correlated with attendance levels. This may be because participants were displeased with the attendance levels, which had a negative impact on the results.

To sum up, it appears that attendance, barriers, and educational objectives are the most crucial factors influencing the effectiveness of the Webex platform.

### 5.1 Comparison with previous studies and novel contributions and insights

The study's focus on the Greek case is important for several reasons. Unlike previous studies that compared the effectiveness of online platforms across different cities globally, this research specifically focuses on Greece. Greece faces unique challenges, such as outdated digital infrastructure and limited IT training among educators, which are not as common in other regions that have been more extensively studied. By examining the Greek educational system, the study brings attention to significant gaps and opportunities for improvement that may not be as apparent in more technologically advanced settings.

Moreover, Greece's comparatively low ranking in the Digital Economy and Society Index (DESI) highlights the importance of improving digital readiness in its educational sector, making this study highly relevant. The Greek case also offers valuable insights into the significance of government support in effectively implementing online education, which is essential for shaping future policies. It highlights how infrastructural deficiencies and lack of official training significantly impacted teachers' effectiveness and student engagement.

Moreover, the research emphasizes the psychological benefits of online education for students in Greece during the COVID-19 pandemic. It offers a comprehensive insight into how online platforms can support the mental well-being of students during emergencies. This analysis, focusing on the Greek situation, contributes to the worldwide discussion on online education by stressing the importance of tailored solutions that address local requirements and obstacles. Consequently, it provides valuable insights that can be relevant to similar scenarios globally.

Regarding novel contributions and insights, this study offers several novel contributions. It provides a detailed, context-specific analysis of the Greek educational system's response to an unprecedented situation, which is relatively underexplored in global studies. It highlights the urgent need for comprehensive IT training programs for teachers to enhance their effectiveness in online teaching. The study also stresses the crucial role of state support and infrastructure in the successful implementation of online education, providing actionable recommendations for future policy improvements. Additionally, it emphasizes the psychological benefits of online education for students, offering a nuanced understanding of how online platforms can support student well-being during crises.

## 6 Conclusions, implications, and recommendations

### 6.1 Objectives and rationale

The objective of the current study was to evaluate the effectiveness of the Webex online platform in Greek secondary education during the COVID-19 lockdown by gathering feedback from secondary education teachers. The study was prompted by the specific challenges faced by Greece, including outdated digital infrastructure and inadequate IT training among educators.

In Greece, there is a significant lack of technological skills and infrastructure. To address this, primary research was conducted to examine the implementation of distance learning in Greek secondary education. The study involved teachers who used the WEBEX platform and were asked to complete a questionnaire. Additionally, a conceptual framework was developed to assess the impact of various factors on the effectiveness of the WEBEX platform.

These challenges were intensified by the sudden transition to online education due to the pandemic. The study aimed to bridge a research gap by providing empirical data on the effectiveness of Webex in Greek secondary education, an area with limited prior research.

### 6.2 Research gap and contribution

Despite numerous studies examining online education's impact during the COVID-19 pandemic, there remains a significant gap in empirical data specifically focused on the Greek secondary education system's use of the Webex platform. This study addresses this gap by providing primary data on the unique challenges and opportunities presented by the rapid transition to distance learning in a technologically underprepared environment like Greece. By highlighting the specific obstacles encountered, such as inadequate IT infrastructure and the psychological benefits for students, this research offers novel contributions to the existing literature.

The study makes a significant contribution by providing a detailed and context-specific analysis of how the Greek educational system responded to an unprecedented situation. It highlights the urgent need for comprehensive IT training programs for teachers and emphasizes the crucial role of state support and infrastructure in successfully implementing online education. Additionally, the study offers actionable recommendations for future policy improvements and emphasizes the importance of tailored solutions that address local requirements and obstacles. These insights have relevance to similar scenarios globally.

### 6.3 Key findings and coefficients calculation

The statistical analysis revealed significant correlations among the study variables.

• **Level of Competency**: Positively correlated with perceived utility (r = 0.45, p < 0.05) and state involvement (r = 0.38, p < 0.05).
• **Perceived Utility**: Positively correlated with state involvement (r = 0.42, p < 0.05).
• **Effectiveness of the Platform**: Negatively correlated with attendance levels (r = −0.30, p < 0.05) and obstacles encountered (r = −0.47, p < 0.05), but positively correlated with the achievement of educational goals (r = 0.52, p < 0.05).

These results indicate that teachers' competency in using the platform and the perceived utility of online education were crucial for its successful implementation. However, the lack of adequate state support and infrastructure posed significant challenges, impacting attendance and the overall effectiveness of the platform. The negative correlation with attendance levels suggests that lower attendance rates were associated with perceptions of reduced effectiveness, highlighting the need for better engagement strategies and policymaking. The positive correlation with the achievement of educational goals underscores the importance of aligning online education practices with clear educational objectives to enhance effectiveness.

It was noted that the infrastructure was inadequate, and there were no options provided for alternative platforms. Even when the attendance rate was high, not all students were able to attend the lessons, which goes against the principles of a catholic education. Furthermore, it seems that the educational goals were not being met, as student interaction and involvement were not at the same levels as when using the previous platform. Lastly, the statistical analysis revealed that attendance, obstacles, and educational goals are the most important factors affecting the effectiveness of the WEBEX platform.

### 6.4 DESI 2019–2022 and Greece's digital progress

In 2022, Greece was ranked 25th out of 27 EU Member States in the Digital Economy and Society Index (DESI). Despite this ranking, Greece has shown significant progress in recent years, indicating that the country is catching up with other EU Member States. Notable improvements include advancements in Very High-Capacity Networks (VHCN) and 5G coverage. However, the adoption of at least 100 Mbps fixed broadband remains low at 9 %, compared to the EU average of 41 % [60]. In terms of digital public services, the number of active e-government users has increased to 69 %, surpassing the EU average of 65 % [60]. The country's level of digital skills is also approaching the EU average, with 52 % of the population having at least basic digital skills. This indicates that the COVID-19 period served as a valuable testing ground for the government to learn lessons.

The "Digital Transformation Bible" of Greece, which was passed into law in 2021, set out the strategic plan for the country's digital advancement in the coming five years. This plan acknowledges the need for digital advancements and encompasses areas such as connectivity, digital skills, digital government, digital business, digital innovation, and the incorporation of digital technology across all sectors of the economy. Furthermore, the "Gov.gr" portal has significantly enhanced the digitalization of public services, leading to a sixfold increase in digital transactions in 2021.

### 6.5 Research results and comparison with DESI 2019

The research findings align with the DESI 2022 results, showing that despite some improvements, Greece still encounters significant challenges in its digital infrastructure. This directly affected the effectiveness of online education during the COVID-19 pandemic. The study emphasized the insufficient IT infrastructure and lack of state support as major obstacles, which is consistent with Greece's lower DESI rankings in 2019 and 2022.

In 2019, Greece was ranked 26th out of 28 EU Member States in the DESI index (UK was still part of EU), which reflected outdated infrastructure and limited digital skills among educators and students. This lack of digital preparedness significantly hindered the transition to online education during the pandemic. This was evident from the study's findings on teachers' IT competency and the effectiveness of the Webex platform. The progress noted in the DESI 2022 report, such as improvements in Very High Capacity Network (VHCN) and 5G coverage, increased digital public service usage, and enhanced digital skills, signals positive developments but also highlights the need for continued efforts to bridge the digital divide [60].

### 6.6 Implications and recommendations

The findings underscore the need for comprehensive training programs for teachers to enhance their IT skills and confidence in using online platforms. Additionally, the state should invest in upgrading the IT infrastructure of schools and provide alternative platforms to avoid reliance on a single system. Addressing connectivity issues and ensuring equitable access to necessary resources for all students is critical to improving attendance and engagement in online education.

The present research has implications for providing feedback on the technological skills and educational effectiveness of a new training method for primary and secondary education in Greece. It has an impact on society and political decisions and could affect the discussion on the necessity, importance, and effectiveness of online/distance learning. The analysis also reveals the needs and shortages concerning teachers' competence and IT systems' effectiveness. However, a limitation of the research is that it only considered the viewpoints of primary and secondary education teachers and did not include students' opinions. Therefore, further research is recommended to include students’ perspectives.

The information and analysis indicate that the Greek educational system was not prepared for the sudden introduction of online teaching, especially for primary and secondary education. Nevertheless, teachers and students did their best, and this situation provided an opportunity to identify deficiencies and areas that need improvement. It is important for the authorities and all stakeholders to understand that technological advancement is a reality and that the educational system needs to be adaptable. Teachers' training, infrastructure updating/upgrading, and students’ education need to be oriented towards the future. The pandemic has provided the opportunity for radical changes, and the Greek state needs to implement such changes quickly and effectively.

### 6.7 Limitations of the research and suggestions for future research

**Limitations of the research.** The period under investigation, from March 2020 until June 2020, indeed presents a constrained timeframe to evaluate the quality of teaching, teacher performance, and the Webex platform's effectiveness during a global pandemic. This brief period, marked by an emergency state of affairs and significant uncertainty, does pose challenges in making robust evaluations. Some points addressing these concerns, include the Emergency Remote Training concept (ERT) and the psycological state of the teachers and students.

The concept of ERT accurately captures the essence of the abrupt transition to online education during the COVID-19 pandemic. ERT is distinct from planned online learning, as it involves the rapid deployment of remote teaching solutions in response to a crisis, without the benefit of proper planning or resources [12]. This context is crucial when evaluating the quality of teaching and the effectiveness of the Webex platform, as the primary goal was continuity of education rather than optimized online learning experiences. The initial section of the study highlights how Greek educational institutions, especialy secondary education, were abruptly forced to adopt online teaching methods, emphasizing the challenges posed by this sudden shift.

Short-term evaluations during the initial months of the pandemic can provide immediate insights into how teachers and students adapted to the crisis. However, these assessments are conducted in the context of emergency response rather than typical educational environments, emphasizing that this study is related to Covid-19. In this context it should be highlighted that long-term studies are essential to understand the full impact and potential improvements over time [11]. The study mentions that evaluations of the Webex platform and online education were conducted during the emergency response period, which may not fully capture long-term effectiveness and adaptation. Still, the use of DESI index research data for comparing the state's digitalization from 2019 to 2022 is essential as it provides a good understanding of the situation and related progress.

The rapid transition to online learning revealed numerous challenges, including inadequate digital infrastructure, lack of teacher training, and varying levels of digital literacy among students and educators [14]. These factors significantly influenced the quality of teaching and learning experiences during the early stages of the pandemic. The research identifies Greece's insufficient digital infrastructure and limited technology skills among secondary education educators as significant obstacles during the transition to online learning.

Furthermore, the psychological state of teachers and students during the pandemic is a critical variable. Stress, anxiety, and the need for social distancing impacted the overall teaching and learning environment [13]. This analysis evaluates these emotional factors, as they significantly impact performance and engagement. The study notes the psychological benefits of online education for students but also highlights the challenges in attendance and achieving educational objectives, suggesting the impact of stress and anxiety.

Despite the constraints, initial results from various studies, including the present one, indicate that teachers and students were able to adjust to online platforms such as Webex to some extent. Teachers' competencies improved over time with experience and available support, and students began to engage with the new learning modalities, albeit with mixed results [17]. The research points out that while some teachers and students managed to adapt, there were significant variations in their experiences and outcomes.

Continued research is vital to comprehensively evaluate the effectiveness of online teaching platforms and methodologies. Longitudinal studies will provide a more accurate picture of how emergency measures can be refined and integrated into regular educational practices [18]. This study concludes by emphasizing the necessity for further research to understand the long-term implications and improvements needed for effective online education.

The short period from March 2020 to June 2020 provides initial insights for an evaluation of teaching quality, teacher performance, and the Webex platform under emergency conditions. Recognizing the unique context of ERT, acknowledging the rapid adaptation challenges, and emphasizing the need for long-term studies are crucial for a comprehensive understanding of these variables.

### 6.8 Future research

Further research should be conducted to encompass the viewpoints of both teachers and students during regular online teaching, rather than just urgent situations, in order to obtain a more comprehensive understanding of the effectiveness of online education.Longitudinal studies could also track the long-term impact of online learning on educational outcomes and psychological well-being. By addressing the identified research gap and providing actionable recommendations, this study contributes to the ongoing discussion on the future of education in a digitally connected world.

## CRediT authorship contribution statement

**Petros Violakis:** Writing – review & editing, Writing – original draft, Visualization, Validation, Supervision, Software, Resources, Project administration, Methodology, Investigation, Funding acquisition, Formal analysis, Data curation, Conceptualization. **Tilemachos Tzakopoulos:** Writing – review & editing, Writing – original draft, Visualization, Validation, Supervision, Software, Resources, Project administration, Methodology, Investigation, Funding acquisition, Formal analysis, Data curation, Conceptualization.

## Data availability

Confidentiality and anonymity are sensitive issues that researchers need to consider carefully. Participants were informed about how their answers would be handled, and no personal data was collected. To protect data, questionnaires were destroyed after they were analyzed.

## Ethics declaration

The study was conducted in accordance with ethical standards and guidelines. All participants provided informed consent before taking part in the study. The research ensured the confidentiality and anonymity of the participants. No personal identifying information was collected; all responses were anonymized. Participants were informed about the purpose of the study, their right to withdraw at any time, and how their data would be used and protected. There were no conflicts of interest reported by the researchers.

## Declaration of competing interest

The authors declare that they have no known competing financial interests or personal relationships that could have appeared to influence the work reported in this paper.
