# Supplementary material for: Teachers' perspectives on the webex online platform for secondary education during the COVID-19 pandemic in Greece
Source: Heliyon. 2024 Oct 22;10(23):e39175. doi: 10.1016/j.heliyon.2024.e39175 (PMC11647793; doi:10.1016/j.heliyon.2024.e39175)
Supplement: Multimedia component 1 [file mmc1.docx]

# **Appendix**

## The research questionnaire

Informative text:

**We kindly invite you to take part in an academic research referring to the effectiveness of online education during the lock-down caused by the COVID-19 pandemic. We value your opinion and hope the present study becomes a reference point for the specific subject, by revealing interest aspects of it. The study is independent, and your anonymity will be preserved. It will take only a few minutes to answer the enclosed questionnaire, while your participation will be valuable. Thank you in advance**

***Please tick the following box, in order to express your willingness to participate in the research.***

***🞏***

***Thank you!***

1. *Demographics:*

| 1.Your Gender | ❒ Woman  ❒ Man |
| --- | --- |
| 2.Your age | ❒ 22-28  ❒ 29-35  ❒ 36-42  ❒ 43- 49  ❒ 49 + |
| 3.years of experience | ❒ 0-5  ❒ 6-10  ❒ 11-15  ❒ 16-20  ❒ 21-25  ❒ 26+ |
| 4.Region | ❒ Attica  ❒ Aegean Islands (North Aegean, South Aegean, Crete)  ❒ Macedonia and Thrace  ❒ Central Greece (Thessaly, Epirus, Sterea Ellada)  ❒ Western Greece (West Greece, Ionian Islands and Peloponnese) |

1. *Questions referring to the level of competence*

| 5. Are you qualified by an official institution on IT subjects? | ❒ 1. Yes  ❒ 2. No |
| --- | --- |
| 6. Were you asked to participate in an evaluation test concerning your competence, before using “webex”? | **❒ 1. Yes**  **❒ 2. No** |
| 7. “I am competent in new technologies and especially internet usage”. *(Please tick on the level of agreement with the above mentioned statement)* | ❒ 1. Totally disagree  ❒ 2. Disagree  ❒ 3. Neither agree, nor disagree  ❒ 4. Agree  ❒ 5. Totally agree |
| 8. “I found it easy to work through the “webex” platform”. *(Please tick on the level of agreement with the above mentioned statement)* | ❒ 1. Totally disagree  ❒ 2. Disagree  ❒ 3. Neither agree, nor disagree  ❒ 4. Agree  ❒ 5. Totally agree |
| 9. “I was even able to help students with the problems they encountered concerning the usage of the platform”. *(Please tick on the level of agreement with the above mentioned statement)* | ❒ 1. Totally disagree  ❒ 2. Disagree  ❒ 3. Neither agree, nor disagree  ❒ 4. Agree  ❒ 5. Totally agree |

1. Questions referring to perceived utility

|  |  |
| --- | --- |
| 10. “The on-line platform was a necessity, so as to continue academic lessons”. *(Please tick on the level of agreement with the above mentioned statement)* | ❒ 1. Totally disagree  ❒ 2. Disagree  ❒ 3. Neither agree, nor disagree  ❒ 4. Agree  ❒ 5. Totally agree |
| 11. “I am willing to continue using the platform, no matter the obstacles, since it helps students stay in touch with the education process”. *(Please tick on the level of agreement with the above mentioned statement)* | ❒ 1. Totally disagree  ❒ 2. Disagree  ❒ 3. Neither agree, nor disagree  ❒ 4. Agree  ❒ 5. Totally agree |
| 12. “I am willing to participate in seminars in order to become more competent because I believe in the utility of distant learning”. *(Please tick on the level of agreement with the above mentioned statement)* | ❒ 1. Totally disagree  ❒ 2. Disagree  ❒ 3. Neither agree, nor disagree  ❒ 4. Agree  ❒ 5. Totally agree |

1. Questions referring to the involvement of the official state

| 13. Did you receive official training on the usage of the platform? | ❒ 1. Yes  ❒ 2. No |
| --- | --- |
| 14. Did you have the potential to contact a “help desk” developed by the ministry of education, in case of trouble? | ❒ 1. Yes  ❒ 2. No |
| 15. “The state provided the necessary infrastructure”. *(Please tick on the level of agreement with the above mentioned statement)* | ❒ 1. Totally disagree  ❒ 2. Disagree  ❒3. Neither agree, nor disagree  ❒ 4. Agree  ❒ 5. Totally agree |
| 16. “The ministry of education and religious affairs provided the necessary guidance for the usage of the platform”. *(Please tick on the level of agreement with the above mentioned statement)* | ❒ 1. Totally disagree  ❒ 2. Disagree  ❒ 3. Neither agree, nor disagree  ❒ 4. Agree  ❒ 5. Totally agree |
| 17. “The ministry of education and religious affairs provided several options concerning alternative platforms”. *(Please tick on the level of agreement with the above mentioned statement)* | ❒ 1. Totally disagree  ❒ 2. Disagree  ❒ 3. Neither agree, nor disagree  ❒ 4. Agree  ❒ 5. Totally agree |

1. Questions referring to attendance levels

| 18. Which was the percentage of attendance, after the first week? | ❒ 1. 0-20%  ❒ 2. 20%- 40%  ❒ 3. 40% - 60%  ❒ 4. 60%- 80%  ❒ 5. 80%-100% |
| --- | --- |
| 19. the percentage of attendance during the period was: | ❒ 1. increasing day-by-day  ❒ 2. decreasing day-by-day  ❒ 3. remaining the same  ❒ 4. fluctuating |
| 20. “I am pleased with the percentage of attendance”. *(Please tick on the level of agreement with the above mentioned statement)* | ❒ 1. Totally disagree  ❒ 2. Disagree  ❒3. Neither agree, nor disagree  ❒ 4. Agree  ❒ 5. Totally agree |

1. Questions referring to obstacles encountered

| 21. “Students could not attend the online courses due to financial constraints” *(Please tick on the level of agreement with the above mentioned statement)* | ❒ 1. Totally disagree  ❒ 2. Disagree  ❒3. Neither agree, nor disagree  ❒ 4. Agree  ❒ 5. Totally agree |
| --- | --- |
| 22. “There were not connectivity issues during the on line courses” *(Please tick on the level of agreement with the above mentioned statement)* | ❒ 1. Totally disagree  ❒ 2. Disagree  ❒3. Neither agree, nor disagree  ❒ 4. Agree  ❒ 5. Totally agree |
| 23. “There were not technological issues that needed troubleshooting during the online courses”. *(Please tick on the level of agreement with the above mentioned statement)* | ❒ 1. Totally disagree  ❒ 2. Disagree  ❒3. Neither agree, nor disagree  ❒ 4. Agree  ❒ 5. Totally agree |
| 24. “Students were on time and entered the platform before and not during the online lesson”. *(Please tick on the level of agreement with the above mentioned statement)* | ❒ 1. Totally disagree  ❒ 2. Disagree  ❒3. Neither agree, nor disagree  ❒ 4. Agree  ❒ 5. Totally agree |

1. Questions referring to the educational goals’ achievement

| 25. “The number of activities and assignments are appropriate, compared to the previous status, so the workload is reasonable” *(Please tick on the level of agreement with the above mentioned statement)* | ❒ 1. Totally disagree  ❒ 2. Disagree  ❒3. Neither agree, nor disagree  ❒ 4. Agree  ❒ 5. Totally agree |
| --- | --- |
| 26. “Selected readings and resources reflect and fit the subject and course learning outcomes, compared to the previous status” *(Please tick on the level of agreement with the above mentioned statement)* | ❒ 1. Totally disagree  ❒ 2. Disagree  ❒3. Neither agree, nor disagree  ❒ 4. Agree  ❒ 5. Totally agree |
| 27. “Students have opportunity for interaction with the content” *(Please tick on the level of agreement with the above mentioned statement)* | ❒ 1. Totally disagree  ❒ 2. Disagree  ❒3. Neither agree, nor disagree  ❒ 4. Agree  ❒ 5. Totally agree |
| 28. “Students’ involvement and activities are adequate in order for them to acquire the knowledge needed” *(Please tick on the level of agreement with the above mentioned statement)* | ❒ 1. Totally disagree  ❒ 2. Disagree  ❒3. Neither agree, nor disagree  ❒ 4. Agree  ❒ 5. Totally agree |

1. Questions referring to the effectiveness of the platform

| 29. “I think the whole project was beneficial for the students, in terms of learning ” *(Please tick on the level of agreement with the above mentioned statement)* | ❒ 1. Totally disagree  ❒ 2. Disagree  ❒3. Neither agree, nor disagree  ❒ 4. Agree  ❒ 5. Totally agree |
| --- | --- |
| 30. “I think the project was beneficial for the students, in terms of emotional support”. *(Please tick on the level of agreement with the above mentioned statement)* | ❒ 1. Totally disagree  ❒ 2. Disagree  ❒3. Neither agree, nor disagree  ❒ 4. Agree  ❒ 5. Totally agree |
| 31. “I am willing to continue with this type of learning”. *(Please tick on the level of agreement with the above mentioned statement)* | ❒ 1. Totally disagree  ❒ 2. Disagree  ❒3. Neither agree, nor disagree  ❒ 4. Agree  ❒ 5. Totally agree |

Thank you very much for your time!
